# Severe Acute Malnutrition Results in Lower Lumefantrine Exposure in Children Treated With Artemether‐Lumefantrine for Uncomplicated Malaria

**DOI:** 10.1002/cpt.1531

**Published:** 2019-07-23

**Authors:** Palang Chotsiri, Lise Denoeud‐Ndam, Elisabeth Baudin, Ousmane Guindo, Halimatou Diawara, Oumar Attaher, Michiel Smit, Philippe J. Guerin, Ogobara K. Doumbo, Lubbe Wiesner, Karen I. Barnes, Richard M. Hoglund, Alassane Dicko, Jean‐Francois Etard, Joel Tarning

**Affiliations:** ^1^ Mahidol‐Oxford Tropical Medicine Research Unit Faculty of Tropical Medicine Mahidol University Bangkok Thailand; ^2^ Epicentre Paris France; ^3^ Epicentre Maradi Niger; ^4^ Malaria Research and Training Centre Faculty of Medicine Pharmacy and Dentistry University of Bamako Bamako Mali; ^5^ Division of Clinical Pharmacology Department of Medicine University of Cape Town Cape Town South Africa; ^6^ WorldWide Antimalarial Resistance Network (WWARN) Oxford UK; ^7^ Centre for Tropical Medicine and Global Health Nuffield Department of Medicine Oxford University Oxford UK; ^8^ Malaria Research and Training Center Faculté de Médecine et d'Odonto‐stomatologie et Faculté de Pharmacie Université des Sciences Techniques et Technologies de Bamako Bamako Mali; ^9^ TransVIHMI UMI 233 Institut de recherche pour le développement (IRD) Inserm U 1175 Montpellier 1 University Montpellier France

## Abstract

Severe acute malnutrition (SAM) has been reported to be associated with increased malaria morbidity in Sub‐Saharan African children and may affect the pharmacology of antimalarial drugs. This population pharmacokinetic (PK)‐pharmacodynamic study included 131 SAM and 266 non‐SAM children administered artemether‐lumefantrine twice daily for 3 days. Lumefantrine capillary plasma concentrations were adequately described by two transit‐absorption compartments followed by two distribution compartments. Allometrically scaled body weight and an enzymatic maturation effect were included in the PK model. Mid‐upper arm circumference was associated with decreased absorption of lumefantrine (25.4% decreased absorption per 1 cm reduction). Risk of recurrent malaria episodes (i.e., reinfection) were characterized by an interval‐censored time‐to‐event model with a sigmoid maximum‐effect model describing the effect of lumefantrine. SAM children were at risk of underexposure to lumefantrine and an increased risk of malaria reinfection compared with well‐nourished children. Research on optimized regimens should be considered for malaria treatment in malnourished children.

**Study Highlights**

**WHAT IS THE CURRENT KNOWLEDGE ON THE TOPIC?**

☑ Artemether‐lumefantrine (Coartem; Novartis, Basel, Switzerland) is the most prescribed antimalarial drug worldwide. However, exposure to lumefantrine is lower in children compared with adults with standard dosing recommendations. Moreover, altered physiological properties in children with severe acute malnutrition (SAM) might reduce drug absorption and further contribute to subtherapeutic exposures. Hitherto, the pharmacology of lumefantrine has been poorly defined in malnourished children, and its population pharmacokinetic (PK) properties have not been studied previously in SAM children. In addition, a lack of PK‐pharmacodynamic (PD) information on antimalarial drugs, when given to malnourished patients, makes it very difficult to dose these children adequately.

**WHAT QUESTION DID THIS STUDY ADDRESS?**

☑ What is the population PK and PD properties of lumefantrine in SAM children?

**WHAT DOES THIS STUDY ADD TO OUR KNOWLEDGE?**

☑ This study is the first population PK‐PD study of lumefantrine in SAM children. Evidently, all malnutrition indicators (i.e., mid‐upper arm circumference (MUAC), weight‐for‐height *z*‐score, and weight‐for‐age *z*‐score) influenced the pharmacological properties of lumefantrine in a similar way. The MUAC was the most significant covariate, resulting in substantially reduced absorption of lumefantrine with increasing malnourishment (25.4% decreased absorption per 1 cm reduction in MUAC). Lower exposure to lumefantrine resulted in an increased risk of acquiring a new *Plasmodium falciparum* infection during the follow‐up period.

**HOW MIGHT THIS CHANGE CLINICAL PHARMACOLOGY OR TRANSLATIONAL SCIENCE?**

☑ This work allows for an in‐depth understanding of the differences in PKs and PDs between SAM children and well‐nourished children. SAM children achieved a significantly lower exposure to lumefantrine than normal children. Lumefantrine dose optimization is needed urgently in this population.

Young children (<5 years of age) are especially vulnerable to malaria, and around 61% of all malaria deaths worldwide occur in this population.^1^ This is of even greater concern in malnourished children, who are at a higher risk of contracting malaria and dying from the disease compared with well‐nourished children.^2^ The World Health Organization (WHO) defines severe acute malnutrition (SAM) by weight‐for‐height *z*‐score (WHZ; <−3), mid‐upper arm circumferences (MUAC; <115 mm), or presence of nutritional edema.^3^ Slower parasite clearance and higher parasite densities have been observed in malnourished children.^4^ Altered physiological properties in malnourished children might change pharmacokinetic (PK) properties (e.g., reduced drug absorption, smaller volume of distribution, reduced plasma concentrations, altered metabolism due to hepatic dysfunction, and reduced drug–protein binding due to hypoalbuminemia).^5^, ^6^

Most clinical studies exclude severely malnourished children resulting in limited evidence on the PKs of drugs in this population, and the few studies available report contradictory results, as illustrated in a recent systematic review.^7^ One study showed a faster quinine clearance, shorter half‐life, and lower quinine concentration 12 hours after dosing with a higher proportion of the metabolite, hydroxyquinine, in global protein‐energy malnourished children when compared with normally nourished children.^8^ However, another study investigating the PKs of quinine after intravenous infusion did not find a significant difference between global protein‐energy malnourished children and well‐nourished children.^4^ Another PK study of orally administered quinine showed lower maximum concentration, longer absorption half‐life, slower clearance, and a longer elimination half‐life in children with kwashiorkor.^9^ For chloroquine, a small study (seven normal and eight undernourished children) did not find any PK differences in the malnourished children.^10^ However, a single‐dose study in children with kwashiorkor found decreased chloroquine absorption, lower chloroquine exposure, and lower peak plasma desethylchloroquine concentration compared with adequately nourished children.^11^

Artemether‐lumefantrine (Coartem; Novartis, Basel, Switzerland) is one of the artemisinin‐based combination therapies recommended by the WHO for the treatment of malaria, and it is the most common antimalarial drug used worldwide.^12^ Artemether is an artemisinin derivative, and it is metabolized rapidly with a terminal half‐life of 2–3 hours to form its active metabolite dihydroartemisinin.^13^ Lumefantrine is eliminated more slowly with a terminal half‐life of 3–6 days, and it is metabolized to desbutyl‐lumefantrine. Both lumefantrine and desbutyl‐lumefantrine possess an *in vitro* antimalarial activity with geometric mean 50% inhibitory concentrations (IC_50_) of 65.2 nM (95% confidence interval (CI): 42.3−101 nM) and 9.0 nM (95% CI: 5.7−14.4 nM), respectively.^14^ Lumefantrine capillary blood concentrations and venous plasma concentrations are highly correlated with no substantial differences in observed *in vivo* concentrations.^15^, ^16^, ^17^ Lumefantrine is highly lipophilic, and its bioavailability increases by 57% (90% CI: 29−56%) when administered together with fat,^18^ and as little as 1.2 g of fat has been shown to maximize the absorption of lumefantrine.^19^ Therefore, artemether‐lumefantrine is recommended to be administered with a fat‐containing meal or a drink (e.g., milk). Irrespectively of drug administration with or without fat, lumefantrine exposure has been reported to be lower in children compared with adults when receiving standard treatment against malaria.^20^ A recently published large pooled PK‐pharmacodynamic (PD) meta‐analysis of artemether‐lumefantrine reported that the dose‐adjusted day 7 lumefantrine concentrations in malnourished young children (aged <3 years with WHZ <−2) was 23% (95% CI: 1−41%) lower than adequately nourished children of the same age and 53% (95% CI: 37−65%) lower than in adults, resulting in increased risk of treatment failure.^21^ The PK and PD properties of lumefantrine have been poorly defined in malnourished children, and its population PK properties have not been studied previously in SAM children. This is the first study aimed to investigate the population PK‐PD properties of lumefantrine in SAM children.

## Results

This clinical trial was an open‐labeled comparative intervention study of artemether‐lumefantrine in 131 SAM and 266 non‐SAM children with uncomplicated *falciparum* malaria, aged between 6 and 59 months. Only 160 of 266 non‐SAM children provided blood samples, and these children were included in the PK analysis. Full demographic characteristics of study participants are presented in **Table** **1**.

**Table 1:** Baseline characteristics of study patients

	PK‐PD arm		PD arm
	SAM	Non‐SAM	Non‐SAM
Total no. of children	131	160	108
Total no. of PK samples	642	700	NA
Total dose of artemether (mg/kg)	17.6 (10.8, 23.7)	11.4 (8.06, 17.6)	13.3 (6.57, 19.4)
Total dose of lumefantrine (mg/kg)	105 (64.8, 142)	68.2 (48.3, 106)	80.0 (39.4, 116)
Total no. of *P. falciparum* reinfection cases	34/131 (23.7%)	61/160 (38.1%)	11/108 (10.1%)
Continuous and categorical covariates at admission			
Age (months)	15 (6.3, 39)	27 (8.0, 53)	24 (7.0, 55)
Axillary temperature at admission (°C)	38.0 (36.8, 39.7)	38.2 (36.8, 39.7)	37.9 (36.2, 40.0)
Number of male patients (%)	50.4% (66/131)	46.9% (75/160)	42.6% (46/108)
Initial parasitemia (no. of parasite/μL)	11,040 (1,092, 154,820)	10,780 (1,121, 152,878)	12,000 (1,058, 123,825)
Anthropometric characteristics			
Body weight (kg)	6.78 (5.07, 10.5)	10.9 (6.84, 15.9)	9.30 (6.20, 15.7)
Height (cm)	73.2 (64.0, 94.2)	85.0 (67.6, 107)	81.0 (66.9, 103)
Body mass index (kg/m^2^)	12.5 (11.0, 13.6)	14.7 (12.8, 17.0)	14.4 (12.3, 17.4)
WHZ	−3.52 (−4.95, −2.60)	−1.07 (−2.58, 0.759)	−1.46 (−3.10, 1.23)
WAZ	−3.40 (−4.46, −2.08)	−1.30 (−3.04, 0.560)	−1.64 (−3.62, 0.308)
HAZ	−1.67 (−2.91, 0.815)	−1.27 (−2.99, 1.56)	−1.31 (−3.15, 1.65)
MUAC (mm)	116 (104, 131)	140 (122, 163)	133 (118, 163)

All values are given as median (95% confidence interval) unless otherwise indicated.

HAZ, height‐for‐age *z*‐score; PD, pharmacodynamic; PK, pharmacokinetic; SAM, severe acute malnutrition; WAZ, weight‐for‐age *z*‐score; WHZ, weight‐for‐height *z*‐score.

### PK model

Lumefantrine capillary plasma concentrations were transformed into their natural logarithms and modeled using nonlinear mixed‐effects modeling. The PK properties of lumefantrine were described best by a two‐compartment disposition model (objective function value difference (ΔOFV) = −753, compared with a one‐compartment disposition model; **Figure S1**). Adding an extra disposition compartment did not improve the model fit significantly (ΔOFV = −5.37). A transit‐absorption model with two transit‐compartments was superior to all other absorption models (ΔOFV = −6.94, compared with first‐order absorption). The absorption rate constant and the transit rate constant were assumed to be equal, thus resulting in no degree of freedom difference to the traditionally used first‐order absorption model. The relative bioavailability of lumefantrine was fixed to unity for the population but allowing for interindividual variability in this parameter improved the model fit substantially (ΔOFV = −262; Box‐Cox transformed distribution provided the best implementation).

**Figure 1:**
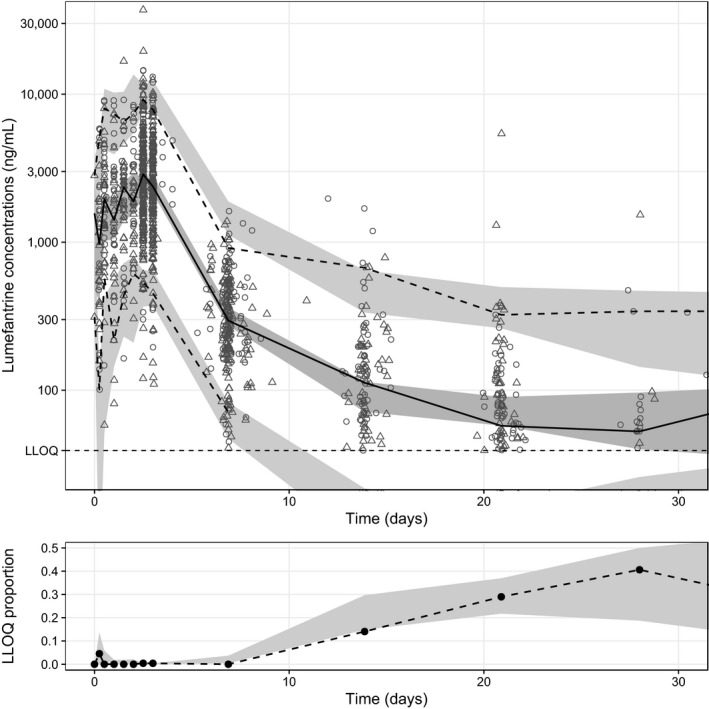
Simulation‐based diagnostics for the final population pharmacokinetic model of lumefantrine. The top panel represents the prediction‐corrected visual predictive check. Open circles represent lumefantrine concentrations in severe acute malnutrition (SAM) children, and open triangles represent lumefantrine concentration in non‐SAM children. The solid line represents the 50^th^ percentile of the observations, and the dashed lines represent the 5^th^ and 95^th^ percentiles of the observations. The gray areas represent the 95% confidence intervals of the simulated percentiles. The horizontal dashed line represents the lower limit of quantification (LLOQ) of lumefantrine (39.1 ng/mL). The bottom panel represents the visual predictive check of the data below the limit of quantification. The dashed line represents the observed proportion of LLOQ samples, and the shaded area represents the simulated 95% prediction interval of the proportion of LLOQ samples.

The fraction of observed concentrations below the lower limit of quantification (LLOQ) were low (84 of 1,341 samples; 6.26%) but evaluated using M1, M3, and M6 methods to avoid possible bias on account of censored data.^22^ Omitting LLOQ data (M1) or using a maximum likelihood approach for LLOQ data (M3) resulted in misspecifications in the fraction of observed LLOQ data. Imputing the first LLOQ data within a patient to be half of the LLOQ value (M6) resulted in a good predictive performance (**Figure** **1**) and was implemented in the final model.

### Covariate model

**Figure 2:**
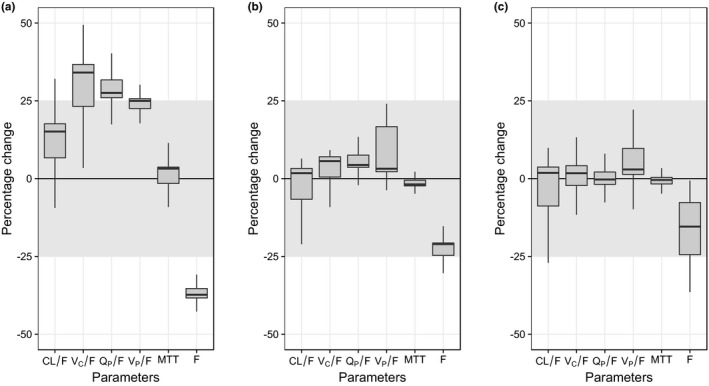
Effect of malnutrition descriptors on the pharmacokinetic (PK) parameters of lumefantrine. The graphs show the relative difference in PK parameter estimates in severe acute malnutrition (SAM) and non‐SAM children (**a**), and change in PK parameters estimates per 1‐cm mid‐upper arm circumferences reduction (**b**), and per 1 weight‐for‐age *z*‐score reduction (**c**). *Y*‐axes represent the change (%) in each PK parameter associated with altered malnutrition status, calculated from 1,000 bootstraps of the full covariate models. The shaded areas represent a covariate effect of ± 25%, assumed to be clinically insignificant. CL/F, oral clearance; F, relative bioavailability; MTT, mean transit time; QP/F, intercompartmental clearance; VC/F, apparent volume of distribution of the central compartment.

Implementation of body weight as a fixed allometric function on all clearance and volume parameters did not improve the model fit (ΔOFV = 66.1), but it was retained in the final model due to the strong biological prior evidence and previously published results.^16^, ^20^, ^23^, ^24^ Other implementations of body weight as a covariate were evaluated (i.e., linear, estimating the exponent in the allometric function, estimating different exponents in the allometric function for SAM and non‐SAM children, and allowing malnutrition measurements to influence the exponent in the allometric function), but none of these demonstrated a substantially improved model fit compared with the fixed allometric function. An enzyme maturation effect on clearance improved the model fit significantly. All investigated indicators of malnutrition (i.e., MUAC, WHZ, and weight‐for‐age *z*‐score (WAZ)) had a significant impact on the relative bioavailability, irrespective of the assumed parameter distribution of the relative bioavailability. Of these three malnutrition covariates, MUAC had the largest drop in ΔOFV (−64.4) and was retained in the final PK model. Addition of any other indicators associated with malnutrition together with MUAC did not result in any additional improvement in model fit. Impact of malnutrition‐associated indicators was further investigated using a full covariate approach. The median bioavailability was 38.6% (95% CI: 74.5−27.4%) lower in SAM children compared with non‐SAM children, and the median bioavailability was reduced by 21.0% (95% CI: 19.5−29.3%) per 1 cm reduction of MUAC or 15.5% (95% CI: 3.07−33.2%) per 1 unit WAZ reduction. Impact of the malnutrition‐associated indicators on other PK parameters was not statistically significant (**Figure** **2**).

The final model showed a satisfactory goodness‐of‐fit (**Figure S1**) with a good predictive performance (**Figure** **1**). Interindividual variability of clearance and volume parameters were estimated close to zero and, therefore, removed in the final model. Eta shrinkages were generally low except for the mean absorption transit time (i.e., 53.2% for mean absorption transit time, 13.2% for bioavailability, and 24.4% for intercompartmental clearance), and epsilon shrinkage was 18.0%. A numerical predictive check (*n* = 2,000) resulted in 1.85% (95% CI: 1.39−3.79%) and 3.01% (95% CI: 1.62−3.48%) of lumefantrine observations being below and above the simulated 95% prediction interval, respectively. Bootstrapping (1,000 resampled datasets) indicated a robust PK model with high precision in parameter estimates. Final primary and secondary PK parameter estimates of lumefantrine are summarized in **Tables** **2** **and** **3**, respectively.

**Table 2:** Parameter estimates from the final population PK and PD model of lumefantrine in SAM and non‐SAM children

	Population estimates^a^ (%RSE)^b^	Bootstrapping median^c^ (95% CI)	%CV of BSV^a^ (%RSE)^b^	Bootstrapping median^c^ (95% CI) for BSV
PK parameters				
F (%)	1 (fixed)	NA	64.0% (7.08%)	63.9% (52.2−76.9%)
Box‐Cox on F	−0.373 (54.5%)	−0.373 (−0.742, 0.205)	NA	NA
MTT (hour)	3.48 (11.7%)	3.48 (2.52, 4.35)	192% (8.88%)	192% (104−259%)
CL/F (L/hour)	2.34 (6.25%)	2.34 (2.20, 2.81)	NA	NA
V_C_/F (L)	110 (4.46%)	110 (101, 123)	NA	NA
Q_P_/F (L/hour)	1.10 (8.10%)	1.10 (0.942, 1.33)	67.8% (7.69%)	67.8% (54.0−82.2%)
V_P_/F (L)	872 (13.9%)	872 (635, 1,200)	NA	NA
σ	0.339 (5.20)	0.339 (0.265, 0.426)	NA	NA
Covariates				
TM_50_ (months)	2.91 (31.5%)	2.91 (2.86, 5.98)	NA	NA
α	1 (fixed)	NA	NA	NA
MUAC on F (% per 1 cm)	25.4% (5.24%)	25.4% (21.3%, 27.1%)	NA	NA
PD parameters				
BASE (reinfections per year)	5.25 (11.8%)	5.58 (4.19, 6.85)	NA	NA
IC_50_ (ng/mL)	156 (12.1%)	156 (141, 214)	NA	NA
γ	4.77 (38.8%)	4.90 (2.30, 9.78)	NA	NA

α, slope factor for enzyme maturation effect; %CV, percentage of coefficient of variation; BASE, baseline hazard rate; Box‐Cox on F, Box‐Cox transformation value of F; BSV, between‐subject variability; CI, confidence interval; CL/F, oral clearance; F, relative bioavailability; IC_50_, lumefantrine 50% inhibitory concentration; MTT, mean transit time; MUAC, mid‐upper arm circumference; NA, not applicable; PD, pharmacodynamic; PK, pharmacokinetic; Q_P_/F, intercompartmental clearance; RSE, relative standard error; SAM, severe acute malnutrition; TM_50_, enzyme maturation half‐life; V_C_/F, apparent volume of distribution of the central compartment; V_P_/F, apparent volume of distribution of the peripheral compartment; σ, residual error variance of lumefantrine concentrations; γ, slope‐factor for the drug effect.

^a^Computed population mean parameter estimates from NONMEM were calculated for a typical individual with a body weight of 9.62 kg. The %CV for the BSV was calculated as $100\times \sqrt{\omega^{2}-1}$. ^b^The %RSEs of the population estimates and BSV were calculated as $100\times \frac{\text{Standard deviation}}{\text{Mean value}}$. ^c^Computed from 1,000 nonparametric bootstraps and presented as 2.5^th^ to 97.5^th^ percentiles of estimates.

**Table 3:** Secondary parameter estimates of lumefantrine in SAM and non‐SAM children

	SAM children (*n* = 131)^a^	Non‐SAM children (*n* = 160)^a^	*P* value^b^
C_max_ (ng/mL)	2,900 (592, 7,180)	3,360 (1,120, 7,750)	0.0007
T_max_ (hour)	5.69 (3.91, 13.1)	5.69 (3.79, 14.4)	0.9214
t_1/2_ (days)	3.10 (1.92, 6.43)	3.54 (1.97, 6.78)	0.0006
Day 7 concentrations (ng/mL)	222 (51.8, 730)	300 (67.5, 798)	<0.0001
AUC_0–28 days_ (hour × μg/mL)	262 (54.4, 661)	316 (78.7, 756)	0.0003

AUC_0–28 days_, area under the concentration‐time curve from time 0−28 days; C_max_, maximum concentration; Day 7 concentrations, lumefantrine capillary blood concentrations at the 7^th^ day after the first dose; SAM, severe acute malnutrition; t_1/2_, terminal elimination half‐life; T_max_, time to maximum concentration.

^a^Median secondary parameter estimates (95% confidence intervals) were obtained from the Bayesian *post hoc* estimates of the final population pharmacokinetic model. ^b^ *P* values were calculated using the Mann–Whitney *U* test.

### PD model

**Figure 3:**
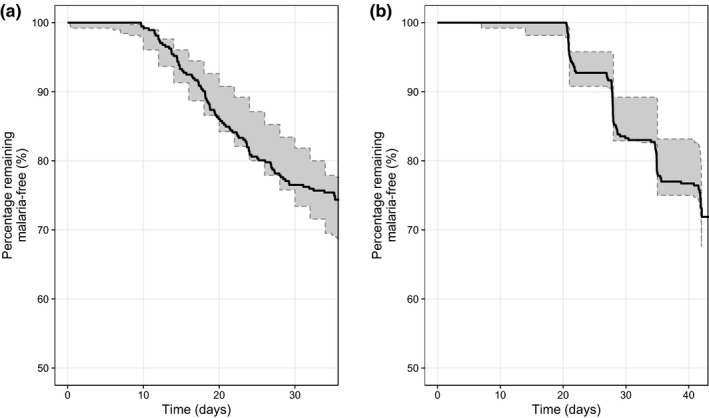
Simulation‐based diagnostics for the interval‐censoring time‐to‐event model of lumefantrine in severe acute malnutrition (SAM) and non‐SAM children. (**a**) Time‐to‐blood stage parasitemia and (**b**) time‐to‐malaria diagnostics. Black solid lines represent the observed Kaplan–Meier plots. Shaded areas represent the simulated 95% prediction intervals from the final pharmacodynamic model.

Observed parasite density at any of the weekly follow‐up visits was back‐extrapolated to the previous malaria‐free visit, assuming an exponential parasite growth. The overall parasite growth rate was estimated to an 11‐fold increase per asexual life cycle (48 hours). Thus, observed recurrent malaria was back‐extrapolated to the starting interval of the blood stage infection (i.e., when parasites emerge from the liver). Children in the PK‐PD arm and PD arm were combined and used for the development of the PD model. Weekly malaria screening identified 95 patients with a reinfection of malaria (median parasitemia: 4,360; 95% CI: 80.0−146,000) who were included in the PD modeling. Four children with recrudescent malaria, three children with *Plasmodium vivax* infections, and 12 children lost to follow‐up or with unidentifiable malaria species were excluded from the PD analysis. Thus, the PD analysis was based on 380 children. The final PK model and individual parameter estimates were fixed and added into the interval‐censoring time‐to‐event model describing the risk of having a reinfection. A sigmoid maximum effect (E_max_)‐function of the predicted lumefantrine concentrations improved the model fit significantly when compared with a model without antimalarial drug effect (ΔOFV = −62.3). No other covariates were found to have a significant impact in the PD model. A visual predictive plot of the interval‐censoring time‐to‐event model exhibited an appropriate predictive performance of time to parasite breakthrough during a malaria reinfection, time to malaria detection, and the recurrent parasite density (**Figure** **3**). Bootstrapping showed robust parameter estimates with acceptable relative standard errors (**Table** **2**).

The *in vivo* minimum inhibitory concentration (MIC) of lumefantrine was estimated based on the predicted lumefantrine concentration at the start of new blood stage infection, using the same back‐extrapolation methodology as described above. The 95^th^ percentile of these predicted lumefantrine concentrations were assumed to be the highest possible concentrations, which still allowed parasite replication. In the patients with recurrent malaria, the predicted MIC values were between 164 and 182 ng/mL, based on the start and the end of the likely time period of new infection emerging from the liver (**Figure S3**).

### *In silico* lumefantrine dose optimization

SAM children had on average 19.2% lower exposure to lumefantrine compared to non‐SAM children in this study, and all children had substantially lower drug exposure compared with adults in previously reported literature.^20^ Based on the final population PK‐PD model developed here, we proposed and evaluated three alternative dosing regimens (i.e., increased, intensified, and extended dosing regimens).

**Figure 4:**
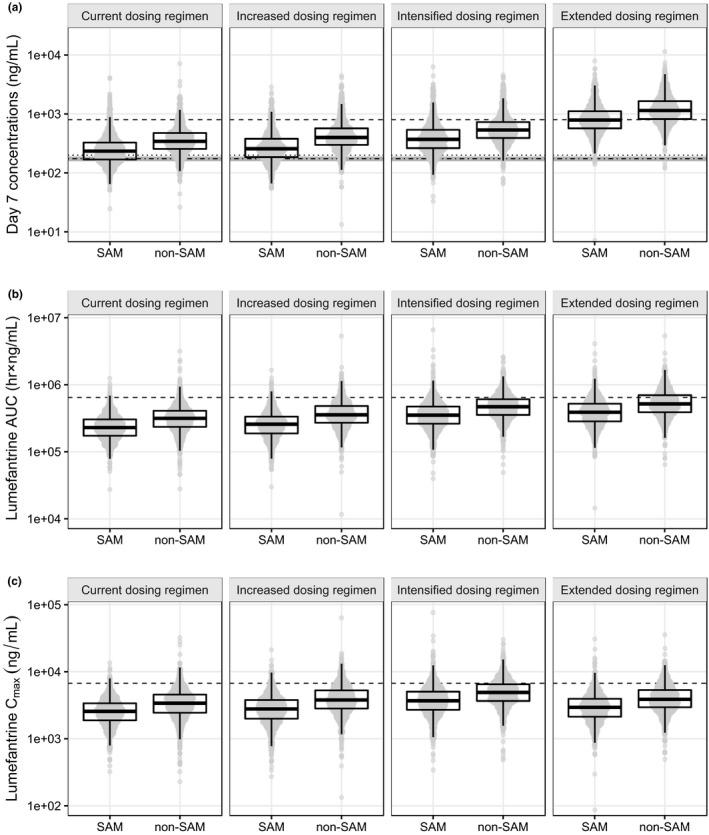
*In silico* lumefantrine dose optimization in severe acute malnutrition (SAM) and non‐SAM children. Predicted day 7 concentrations (**a**), area under the concentration‐time curve (AUC) (**b**), and maximum concentrations (C_max_) (**c**) of lumefantrine were plotted. Boxes and whiskers represent the median with interquartile range and the 95% prediction intervals, respectively. Horizontal dashed lines in panel **a** represent the median day 7 lumefantrine concentration after standard dosing regimen in nonpregnant adult patients (801 ng/mL).^20^ The dotted (200 ng/mL) and dashed‐dotted (175 ng/mL) lines in panel **a** represent the previously defined day 7 lumefantrine concentrations associated with therapeutic efficacy.^21^, ^41^ Gray areas in panel **a** represent the predicted clinical minimum inhibitory concentration value between 164 ng/mL and 182 ng/mL. Horizontal dashed lines in panels **b** and **c** represent the median lumefantrine exposure (AUC; 647,025 hour × ng/mL) and C_max_ (6,731 ng/mL) after standard dosing in nonpregnant adult patients.^20^

PK exposure parameters (i.e., area under the concentration‐time curve (AUC), peak plasma concentration (C_max_), and day 7 concentration) of the increased dose regimen were approximately equivalent to standard dosing, because of a relatively lower bioavailability of the increased dose (i.e., approximately half of that seen in children receiving standard dose). However, both the intensified and extended dosing regimens were able to increase the exposure to lumefantrine in SAM children to similar levels as that seen in non‐SAM children receiving standard dosing (**Figure** **4**, **Figure S4**). Nevertheless, lumefantrine exposure was overall low in children compared with adults, resulting in 98.4% of SAM children and 94.7% of non‐SAM children having lower exposure than the reported median exposure in adult patients.^20^ The increased dose regimen exhibited negligible improvement in children because of dose‐limited absorption. The intensified and extended dosing regimens resulted in 76.9% and 69.1%, respectively, of non‐SAM children having a predicted exposure below median values reported in adults. A smaller improvement was seen in SAM children after intensified and extended dosing, resulting in 92.7% and 89.3%, respectively, of children having a predicted lower exposure compared with median values reported in adults.

Therapeutic efficacy could not be evaluated because only four patients presented recrudescent malaria in either group during the 42 days of follow‐up. However, time above MIC should be highly correlated to the risk of therapeutic efficacy because residual lumefantrine concentrations above the MIC value eliminate residual parasites to avoid recrudescent infections. Time above MIC was 8.05 (95% CI: 5.62−28.9) days and 9.33 (95% CI: 6.60−39.3) days for SAM and non‐SAM children, respectively, after standard dosing. Time above MIC in SAM children could be expanded to 9.81 (95% CI: 6.67−50.8) days and 12.3 (95% CI: 8.10−52.0) days after the intensified and extended dosing regimens, respectively.

PD protective efficacy of different dosing regimens was evaluated by simulating the total incidence of malaria reinfections during the 42 days of follow‐up (**Figure S5**). PD simulations using standard dosing predicted similar protective efficacy toward reinfections after artemether‐lumefantrine treatment in SAM and non‐SAM children (64.5% (95% CI: 57.7−71.5%) vs. 67.3% (95% CI: 59.2−74.3%), respectively, being malaria‐free at day 42). The increased dose regimen did not improve the protective efficacy of artemether‐lumefantrine because of similar exposure to lumefantrine. A moderate improvement in protective efficacy could be seen with intensified and extended dosing regimens (**Table S1**).

## Discussion

Comorbidity of malaria and malnutrition is common, reaching high prevalence in the Sahel region where acute malnutrition episodes among children <5 years of age occur frequently during the malaria season. The review of the literature does not provide a clear picture of the effect of malnutrition on the malaria risk, but some studies show that malnutrition increases mortality due to malaria in children <5 years of age.^7^ To our knowledge, this study comparing the PKs and PDs of lumefantrine in a population of malnourished and non‐malnourished children <5 years of age is the largest cohort reported in the literature to date. We demonstrated that drug exposure in children correlated with risk of reinfection. All investigated indicators of severe acute malnutrition (i.e., MUAC, WHZ, and WAZ) had a significant impact on the absorption of lumefantrine. MUAC was significantly associated with decreased relative bioavailability of lumefantrine (25.4% decrease per 1‐cm reduction).

The PK properties of lumefantrine were explained by a two‐distribution compartment model, which is similar to what has been reported previously.^24^ Delayed lumefantrine absorption has been reported previously,^25^, ^26^ and the present data were best described by a two‐transit compartment absorption model to mimic this delay in drug absorption along the gastrointestinal tract.^20^, ^27^ The absolute bioavailability cannot be estimated using oral data alone, but it allows for estimating the relative difference in bioavailability between patients (i.e., interindividual variability). A Box‐Cox transformed distribution of the relative bioavailability was implemented in the final model, which is identical to previously published findings.^20^ The estimated negative shape parameter of the Box‐Cox distribution indicated a left‐skewed distribution of the absorption parameter, which is likely to be explained by the dose limited absorption of lumefantrine.^19^, ^20^ Several PK studies have characterized the PK properties of both the lumefantrine and the metabolite desbutyl‐lumefantrine.^23^, ^25^, ^27^, ^28^ However, in the present study, the metabolite was not measured and only the PKs of the parent drug were analyzed.

Even though only 6.26% of samples were below the LLOQ, different methods were evaluated to incorporate these samples.^29^ LLOQ data were omitted (M1 method) in a previous publication, but we evaluated the M1, M3, and M6 methods in the present analysis to avoid any potential censoring bias. Indeed, the predicted fraction of samples below the LLOQ was underpredicted using both the M1 and the M3 method. However, the M6 method showed a good predictive performance, as illustrated in **Figure** **1** and was used in the final model.

Allometrically scaled body weight on all clearance and volume parameters, and an enzyme maturation effect were included in the final model due to prior knowledge and a strong biological support.^20^, ^30^ Covariates associated with severe acute malnutrition (MUAC and WHZ) and severe underweight (WAZ) were investigated both with a stepwise approach and a full covariate approach. All three of these were highly correlated and affected the PK parameters in the same direction (**Figure** **2**), both in the stepwise approach and in the full covariate approach, probably due to the strong correlation between these covariates (**Figure S2**). Both of these analyses suggested that the bioavailability is reduced with an increase in malnutrition severity. The stepwise covariate modeling showed that MUAC (on the relative bioavailability) was the most significant covariate and was retained in the final model. Artemether‐lumefantrine was administered together with milk (2.5 g of fat) for non‐SAM children and together with ready‐to‐use therapeutic food (RUTF; 32.9 g of fat) to SAM children, and a small amount of fat (1.2 g) has been shown to enhance the exposure of lumefantrine up to 90% compared with when administered to fasting subjects.^19^ Thus, the administered fat content should maximize the absorption of lumefantrine in both SAM and non‐SAM children. Therefore, the coadministration of RUTF to SAM children should guarantee an optimal absorption and excludes this as an explanation why SAM children had a lower bioavailability. Several reasons could explain lower bioavailability in SAM children and several physiological changes have been identified in the gastrointestinal tract in malnourished individuals, for example, anorexia, vomiting, diarrhea, hypochlorhydria, mucosal atrophy, delayed gastric emptying, pancreatic dysfunction, and alterations in the intestinal micro‐ecology.^5^, ^6^

Reinfections with *P. falciparum* malaria were used as the PD end point, in order to describe the protective effect of lumefantrine. Only a few patients showed recrudescent malaria (i.e., treatment failure), and they were, therefore, excluded from the PD analysis. In addition, *P. vivax* infections have a different mechanism compared to *P. falciparum* infections, and these patients were excluded from the analysis. Both the PK‐PD and the PD arms were included into the PD data analysis and modeled simultaneously to maximize the amount of data. Commonly, time‐to‐event models are used to describe the time to malaria detection and to quantify the impact of drug concentration by linking the PK model with a PD E_max_‐model. However, the estimated IC_50_ in such models will underpredict the true IC_50_ value because the model assumes that the recurrent malaria appears exactly on the day of the follow‐up visit. In reality, the blood stage infection started several days before the time of microscopy detection and the parasites have managed to grow through whatever drug concentrations that were present at that time. Bergstrand *et al*. and Chotsiri *et al*.^31^, ^32^ suggested that the PD model should be based on the possible starting time interval of the malaria erythrocytic stage by back‐extrapolating the measured parasite density at malaria re‐infection using the parasite growth rate. The interval censoring time‐to‐event model should then be able to estimate an IC_50_ closer to the true value. Therefore, the present study took this into account by determining the likely time of the emergence of the blood stage malaria infection and used this time interval as the event time. Observed parasite density at reinfection and the individual microscopic detection limit were used to determine the individual parasite growth rate. However, the average or median of estimated individual growth rates will not represent the true growth rate in the population because an estimated slow parasite growth rate might be a result of a recent infection or a lower parasite density than the individual detection limit at the previous malaria‐free visit. Therefore, it is expected that the true population value is skewed toward the higher estimated growth rates. Thus, the overall parasite growth rate in the population was defined by the 95th percentile of individually estimated parasite growth rates, resulting in an 11‐fold increase in parasite densities per asexual reproductive cycle (48 hours). This value is close to what would be expected from historical data.^33^, ^34^ By modeling the starting time of the erythrocytic stage, the lumefantrine concentrations at the time of the emerging *P. falciparum* parasites were predicted, and, thus, provided insight into the clinical MIC values in this population. The predicted MIC values were between 164 and 182 ng/mL, which is similar to previously reported cutoff day 7 values associated with therapeutic efficacy.

The visual predictive check of the PK‐PD model (**Figure** **4** **b**) supported the interval‐censoring time‐to‐event model and showed a good prediction of the time to malaria detection. The final PD model and parameter estimates, including IC_50_, from this study were similar with a previous report studying Thai women infected with *P. falciparum*.^25^ The baseline hazard in the present study was slightly higher, probably because this study was conducted in an area with higher endemicity, whereas the previous study was conducted in a low transmission area (Thailand). Malnutrition was not a significant covariate in the PD model, probably because it was already incorporated in the PK model.

This study showed that malnourishment had a dramatic effect on the absorption of lumefantrine when given to children. This threatens our ability to treat malaria in this group, as it will result in inadequate exposure to lumefantrine in SAM children. Alternative dosing regimens were evaluated using *in silico* dose optimization. Due to the dose limited absorption of lumefantrine, an increased dose regimen could not compensate fully for the lower exposure observed in SAM children.^20^ However, both the intensified regimen (thrice daily for 3 days), and the extended regimen (twice daily for 5 days) of artemether‐lumefantrine in SAM children resulted in equivalent exposures in non‐SAM and SAM children (**Figure** **4**). This resulted in an expanded time above MIC and slightly higher protective efficacy (**Figure S4** **,** **Table S1**).

In conclusion, the population PK properties of lumefantrine in this study were successfully explained by a two‐compartment disposition model with two transit‐compartments in the absorption phase. Body weight as an allometric function and age as an enzyme maturation effect were included into the PK model. Malnutrition had a significant impact on the absorption of lumefantrine, resulting in substantially lower drug exposure with increasing malnutrition. A parasitemia‐corrected time‐to‐event model was developed to explain the post‐treatment prophylactic effect of lumefantrine against malaria reinfections. Research on altered dosing regimens should be considered for optimal treatment of malaria in malnourished children.

## Methods

### Study design

An open comparative intervention study was conducted to determine the clinical efficacy and PK‐PD properties of lumefantrine in African SAM (*n* = 133) and non‐SAM children (*n* = 266). A subset of the whole study was chosen to be part of the PK study (*n* = 131 for SAM children and *n* = 160 for non‐SAM children). Details on the study protocol as well as the clinical efficacy and safety have been published previously.^35^, ^36^ This study was conducted at two hospitals, the Oulessebougou District Hospital, Koulikoro region, Mali, and at the primary healthcare center on Andoume, Maradi City, Niger. Two identical versions of the protocol were prepared, a French protocol that was approved by the Ethics Committee of the Faculty of Medicine and Odonto‐Stomatologie and the Faculty of Pharmacy in Bamako, Mali (number 2O13/93/CE/FMPOS) and Niger National Ethics Committee of the Ministry of Health (number 004/2014/CCNE), and an English version, which was approved by the MSF Ethical Review Board. The study was registered at Clinicaltrial.gov (registration number: NCT01958905, registration date: October 7, 2013). Only children whose parents or guardians provided a written informed consent were enrolled in this study.

Children aged between 6 and 59 months with uncomplicated *falciparum* malaria were eligible for enrollment in this study. According to the WHO criteria of SAM, children with WHZ < −3 and/or MUAC < 115 mm were classified as SAM. Two misclassified children without the SAM condition were later enrolled to the non‐SAM group. Children with kwashiorkor, severe stunting (severe chronic malnutrition, height‐for‐age *z*‐score < −3), severe anemia, known underlying or chronic diseases, and other complications requiring hospitalization were excluded from the study.

Fixed‐dose combination tablets of nondispersible artemether 20 mg and lumefantrine 120 mg (Coartem) were given according to the weight‐based manufacturer recommended dose (1 tablet < 15 kg and 2 tablets 15–25 kg), twice daily for 3 days. Study drugs were administered with fat (i.e., one glass of milk (~ 250 mL containing 2.5 g of fat) in the non‐SAM group, and RUTF; Plumpy'Nut, Société de Transformation Alimentaire (STA), Niamey, Niger one bag of 92 g containing 32.9 g of fat) in the SAM group. If the children vomited within 30 minutes of dose administration, a repeat dose was administered. If the children vomited after the second dose, they were given rescue oral medication (artesunate‐amodiaquine fixed‐dose formulation; ASAQ Winthrop, Geneva, Switzerland) and were excluded from the study.

### Drug quantification

A population‐based sparse sampling approach was used to limit the number of PK samples required per child.^37^ For each child, five capillary blood (50 μL) samples were collected; the first at one of the following randomly allocated times: 6, 12, 24, 36, or 48 hours, the second at hour 60, the third at hour 72, the fourth at day 7, and the fifth at either day 14 or day 21 (randomly allocated) post‐treatment initiation. Each blood sample was transferred onto pretreated (0.75 M tartaric acid) filter paper (Whatman 31 ET Chr). The filter paper spots were left to dry unaided at room temperature and then sealed in individual plastic bags with desiccant and stored at room temperature away from heat and excessive light until sent to the Division of Clinical Pharmacology, University of Cape Town, South Africa, for drug measurement. Dry blood spot concentrations of lumefantrine were measured using solid phase extraction followed by liquid chromatography coupled with tandem mass spectrometry. Quality control samples at low, medium, and high concentrations (100, 4,000, and 8,000 ng/mL, respectively) were analyzed in duplicate within each batch of study samples to ensure accuracy and precision of the drug assay. The combined accuracy was between 96.2% and 105%, and precision (coefficient of variation (%CV)) between 1.80% and 10.6%, for the low, medium, and high quality controls, respectively. The LLOQ of the assay was set to be 39.1 ng/mL.

### PK‐PD analysis

Lumefantrine capillary blood concentrations were transformed into their natural logarithms and analyzed using nonlinear mixed‐effects modeling in NONMEM version 7.3 (Icon Development Solution, Ellicott City, MD). Pirana version 2.9.0,^38^ Perl‐speaks‐NONMEM version 4.6.0 (PsN),^39^ and Xpose version 4.0,^40^ were used for automation, model evaluation, and diagnostics during the model building process. The final PK model was fixed and individual estimates imputed into the exposure‐response model. Time to malaria reinfection during the 42‐days of follow‐up was described using an interval‐censoring time‐to‐event model. PD data were modeled using the Laplace estimation method with interactions. Biologically plausible covariates (i.e., SAM status, WAZ, WHZ, height‐for‐age z‐score, MUAC, age, body mass index, and body weight) were evaluated in the PK and PD models. Details of the data analyses, model diagnostics, and *in silico* lumefantrine dose optimization can be found in the **SI Methods**.

## Funding

The Mahidol‐Oxford Tropical Medicine Research Unit is supported by the Wellcome Trust of Great Britain. The pharmacokinetic and pharmacodynamic analysis was supported by a grant from the Bill and Melinda Gates Foundation to Professor Joel Tarning. The funding bodies did not have any influence on the collection, analysis, interpretation of data, writing of the manuscript, or in the decision in submitting the manuscript for publication.

## Conflict of Interest

The authors declared no competing interests for this work.

## Author Contributions

P.C., R.M.H., and J.T. wrote the manuscript. L.D.N., E.B., O.G., O.K.D., K.I.B., A.D., and J.F.E. designed the research. L.D.N., E.B., O.G., H.D., O.A., M.S., P.J.G., and O.K.D. performed the research. P.C. and J.T. analyzed the data. L.W. and K.I.B. contributed new reagents/analytical tools.

## Supporting information

Supplementary methods
Figure S1
Figure S2
Figure S3
Figure S4
Figure S5
Table S1
Supplementary references
