# Supplementary material for: Severe Acute Malnutrition Results in Lower Lumefantrine Exposure in Children Treated With Artemether‐Lumefantrine for Uncomplicated Malaria
Source: Clin Pharmacol Ther. 2019 Jul 23;106(6):1299–309. doi: 10.1002/cpt.1531 (PMC6896236; doi:10.1002/cpt.1531)
Supplement: Supplementary file 1 — Supplementary methods Figure S1 Figure S2 Figure S3 Figure S4 Figure S5 Table S1 Supplementary references [file CPT-106-1299-s001.pdf]

Severe acute malnutrition results in lower lumefantrine exposure in children treated with artemether-lumefantrine for uncomplicated malaria

SUPPLEMENTARY METHODS

Population pharmacokinetic analysis

Lumefantrine capillary blood concentrations were transformed into their natural logarithms and analysed using a nonlinear mixed-effects modelling approach in NONMEM version 7.3 (Icon Development Solution, Ellicott City, MD). Pirana version 2.9.0 (1), Perl-speaks-NONMEM version 4.6.0 (PsN) (2), and Xpose version 4.0 (3), were used for automation, model evaluation, and diagnostics during the model building process. The first-order conditional estimation method with interactions (continuous data only) or the Laplacian estimation method (combination of continuous and categorical data) was used throughout the pharmacometric analysis. Data below the LLOQ were either omitted (M1-method), incorporated by imputing the first LLOQ data as half of LLOQ (M6-method) or modelled as categorical data (M3-method) (4).

One-, two-, and three-disposition compartment models were evaluated to describe the lumefantrine capillary blood concentration-time profile. The best performing disposition model was used to evaluate absorption characteristics of lumefantrine (i.e. first-order absorption with and without lag-time, zero-order absorption, and transit-absorption). For the transit absorption model, the absorption constant (K_a) and transit rate constant (K_{tr}) were assumed to be equal (5).

Pharmacokinetic parameters were assumed to be log-normally distributed and therefore implemented as an exponential between-subject variability as follows:

$$\theta_i = \theta \times \exp(\eta_{i,\theta})$$

where Θ_i is individual ith pharmacokinetic parameter estimate, Θ is the typical parameter estimate of the population, and $\eta_{i,\theta}$ is the between-subject variability of parameter Θ and the ith individual, assumed to be normal distributed with zero mean and variance ω^2. Estimated between-subject variability below 10% was fixed to zero. Relative bioavailability was fixed to unity for the population, but allowed for estimation of inter-individual variability in the same parameter. A Box-Cox transformed distribution was applied for the implementation of relative bioavailability, as described below (6):

$$\eta_{i,\mathrm{Transformed}} = \frac{\left(\exp(\eta_{i,F})\right)^{\theta_{\mathrm{Box-Cox}}} - 1}{\theta_{\mathrm{Box-Cox}}}$$

$$F_i = \theta_F \times \exp(\eta_{i,\mathrm{Transformed}})$$

where $\eta_{i,\mathrm{Transformed}}$ is the transformed distribution of the inter-individual variability, $\eta_{i,F}$ is the normal distribution of bioavailability, $\theta_{\mathrm{Box-Cox}}$ is the Box-Cox shape parameter, θ_F is the typical value of relative bioavailability (fixed to 1), and F_i is the individually estimated relative bioavailability. Residual unexplained variability was modelled as an additive error on the log-transformed observed concentrations (equivalent to an exponential error on an arithmetic scale).

Individual body-weight (BW_i) was introduced into the pharmacokinetic model as a fixed allometric function on all volume (exponent of n = 1.00) and clearance (exponent of n = 0.75) parameters, scaled to the median body weight (9.62 kg) of the study population as follows:

$$\theta_i = \theta \times \exp(\eta_{i,\theta}) \times \left(\frac{\mathrm{BW}_i}{9.62}\right)^n$$

This covariate was introduced into the model *a priori* based on the strong biological prior of this covariate relationship, and it was demonstrated to have a significant impact in a large pooled meta-analysis of the pharmacokinetic properties of lumefantrine (7). However, other implementations of body weight as a covariate were evaluated during model building to assess possible alterations in the allometric function due to malnutrition (i.e. linear, estimating the exponent in the allometric function, estimating different exponents in the allometric function for SAM and non-SAM children, and allowing malnutrition measurements to influence the exponent in the allometric function).

All other covariates were investigated by using a stepwise forward inclusion (p-value < 0.05), followed by stepwise backward elimination (p-value > 0.001) (8). Thus, a strict p-value of 0.001 was used in the backward elimination for a parsimonious modelling approach in order to retain covariates in the final model. A maturation process of enzyme-dependent metabolic elimination was evaluated on the elimination clearance of lumefantrine as follows (9):

$$\mathrm{MF} = \frac{\mathrm{AGE}^{\alpha}}{\mathrm{TM}_{50}^{\alpha} + \mathrm{AGE}^{\alpha}}$$

$$\mathrm{CL}_i = \theta_{\mathrm{CL}} \times \exp(\eta_{i,\mathrm{CL}}) \times \left(\frac{\mathrm{BW}_i}{9.62}\right)^{0.75} \times \mathrm{MF}$$

where MF is the maturation factor, AGE is the age of the child, α is a shape factor, TM_{50} is the maturation half-life, CL_i is the individually estimated elimination clearance, θ_{CL} is the typical (population) value of the elimination clearance, and $\eta_{i,\mathrm{CL}}$ is the between-subject variability of clearance for individual i.

Malnutrition parameters (weight-for-age z-score; WAZ, weight-for-height z-score; WHZ, and height-for-age z-score; HAZ) were calculated according to the WHO guideline (10). Severe acute malnutrition status was defined as the WFH z-score less than -3, and/or the mid-upper arm circumference (MUAC) less than 115 mm. All continuous and categorical covariates (sex, BMI, MUAC, body temperature at admission, haemoglobin level, HZA, WAZ, WHZ,

BMI-for-height z-score, and SAM status) were investigated by using a stepwise approach as described above. The effect of malnutrition status was also modelled separately using a full covariate approach in which the MUAC, WAZ, and WHZ were implemented as a continuous covariate on all pharmacokinetic parameters in the final pharmacokinetic model. Any pharmacokinetic parameter estimates changing more than ±25% due to malnutrition was deemed as a clinically relevant change. Secondary pharmacokinetic parameter estimates were derived from the *post hoc* pharmacokinetic parameter estimates of the final pharmacokinetic model.

Population pharmacodynamic analysis

The final pharmacokinetic parameter estimates were fixed and the pharmacokinetic model implemented in the pharmacodynamic time-to-event model. Time-to-malaria reinfection during the 42-days of follow-up was described using an interval-censoring time-to-event model. PCR genotyping at enrolment and at the day of recurrent malaria was performed to determine if it was a reinfection or a recrudescence. Only *P. falciparum* reinfections were included in the pharmacodynamic analysis. Recrudescent malaria, reinfections with other *Plasmodium* species, and patients lost to follow-up were excluded from the pharmacodynamic analysis. Nonlinear mixed-effects modelling with the Laplace estimation method with interactions was applied throughout pharmacokinetic-pharmacodynamic modelling.

The lumefantrine protective effect ($\mathrm{LF_{EFF}}$) was defined by a sigmoid $\mathrm{E_{MAX}}$-function, where E_{MAX} is the maximal drug effect, $\mathrm{CP}(t)$ is the model predicted lumefantrine capillary blood concentration at time t, $\mathrm{IC_{50}}$ is the lumefantrine capillary blood concentration needed in order to reduce the hazard of malaria reinfection by 50%, and γ is the shape parameter according to the following equation:

$$\mathrm{LF_{EFF}} = 1 - \frac{E_{\mathrm{MAX}} \times \mathrm{CP(t)}^{\gamma}}{\mathrm{IC_{50}^{\gamma}} + \mathrm{CP(t)}^{\gamma}}$$

A constant baseline hazard of malaria infection was assumed and the hazard function ($\mathrm{Hz}(t)$) was defined by multiplication of the constant baseline hazard (θ_{BASE}) and lumefantrine drug effect ($\mathrm{LF_{EFF}}$). The survival function ($S(t)$) was defined as the exponent of the cumulative hazard. The probability density function for acquiring a malaria reinfection at a specific time-point ($P(t)$) was defined as the multiplication of the hazard function with the survival function.

$$\mathrm{Hz}(t) = \theta_{\mathrm{BASE}} \times \mathrm{LF_{EFF}}$$

$$S(t) = \exp\left(\int_0^t \mathrm{Hz}(t)dt\right)$$

$$P(t) = S(t) \times \mathrm{Hz}(t)$$

The lag-time between an emerging blood stage infection from the liver and the microscopy detection of malaria is dependent on several factors, i.e. parasite growth rate, number of parasites emerging from the liver, and drug concentrations. Therefore, the likely time interval of an emerging blood stage infection was back-extrapolated using the observed number of parasites ($\mathrm{PAR_{OBS}}$) at the time of microscopic detection with a fixed exponential parasite

growth rate (k_{growth}). The fixed parasite growth rate was based on individual parasite growth rates estimated using the study data. Individual parasite growth rates were calculated using the observed parasite density at the time of microscopy detection and the individually adjusted microscopy detection limit, assuming a parasite density at the detection limit at the previous malaria-free visit. The parasite growth rate in the population was fixed to the 95th percentile of the individual growth rates since the high values are more likely to reflect a true estimate of the growth rate (i.e. individually predicted slow growth rates can be a result of recent infections and/or parasite densities below the detection limit at the previous malaria-free visit). The estimated number of emerging blood stage parasites was assume to range between 10^4 and 10^5 parasites (11, 12). Therefore, the time interval of emerging blood stage parasites starting from low initial blood stage parasitaemia (I_{start} at 10^4) to high initial parasitaemia (I_{end} at 10^5), was calculated according to the following equations:

$$I_{\text{start}} = \frac{1}{k_{\text{growth}}} \times \ln\left(\frac{\text{PAR}_{\text{OBS}}}{10^4}\right)$$

$$I_{\text{end}} = \frac{1}{k_{\text{growth}}} \times \ln\left(\frac{\text{PAR}_{\text{OBS}}}{10^4}\right)$$

The final pharmacodynamic model was based on an interval-censoring time-to-event approach, accounting for the likely interval of emerging blood stage infections. Thus, the probability of a red blood cell infection within this interval ($\Pr(a < t < b \mid \theta)$) and the probability of a malaria re-infection occurring after the follow-up period was defined as follows:

$$\Pr(a < t < b \mid \theta) = 1 - [S(I_{\text{end}}) - S(I_{\text{start}})]$$

$$\Pr(t > T \mid \theta) = S(T)$$

Biologically plausible covariates (i.e. SAM status, WFH, WFA, MUAC, age, BMI, and body weight) were evaluated with a stepwise approach as linear and exponential functions on baseline hazard in the final time-to-event model as for the pharmacokinetic model. Forward inclusion criteria using p-value cut-off of 0.05, and the backward elimination criteria using a stringent p-value of 0.001. Additionally, in vivo minimum inhibitory concentrations (MIC) of lumefantrine was estimated based on the individually predicted lumefantrine concentrations at the start of the blood stage infections. To avoid any potential impact of outliers, the 95th percentile of individually predicted MIC values was calculated and presented as the clinical MIC.

Model diagnostics

Model fit was evaluated primarily by the objective function value (OFV; calculated by NONMEM as proportional to -2 × log-likelihood of the data). Model discrimination between two hierarchical models was determined by a likelihood ratio test, based on the Chi-square distribution of the OFV (i.e. p-value < 0.05 corresponding to ΔOFV > 3.84, at 1 degree of freedom difference). Potential model misspecification and systematic errors were evaluated by basic goodness-of-fit diagnostics. Eta and epsilon shrinkages were used to assess the ability of detecting model misspecifications in goodness-of-fit diagnostics (13). Model robustness and non-parametric confidence intervals were

evaluated by bootstrap diagnostics (n=1,000). Predictive performances of the final models were illustrated by prediction corrected visual and numerical predictive checks (n = 2,000) (14). The 5th, 50th, and 95th percentiles of the observed concentrations were overlaid with the 95% confidence intervals of each simulated percentile to detect model bias.

In silico lumefantrine dose optimisation

The final pharmacokinetic-pharmacodynamic model was used to simulate lumefantrine exposures and treatment outcomes in SAM and non-SAM children. Alternative dosing strategies included: (1) increased dose regimen (adding 1 extra tablet of 20 mg artemether and 120 mg lumefantrine to the standard dose given twice daily for three days), (2) intensified dosing regimen (the standard dose given thrice daily for three days), and (3) extended dosing regimen (the standard dose given twice for five days). Lumefantrine exhibit dose-dependent absorption and a saturation model was implemented on the relative bioavailability, according to previously published results (7), in order to avoid bias in dose optimisation simulations.

$$F_i = \theta_F \times \exp(\eta_i) \times \left(1 - \frac{\text{Dosage}}{\theta_{\text{Dosage}_{50}} + \text{Dosage}}\right)$$

The $\theta_{\text{Dosage}_{50}}$ was fixed to 3.86 mg/kg according to previous literature (7).

Day-7 concentrations are associated with therapeutic success and have been used previously as a pharmacokinetic endpoint. However, different studies have suggested different cut-off levels of day 7 lumefantrine venous plasma concentrations, ranging from 175 ng/mL (15), 200 ng/mL (16, 17) to 280 ng/mL (18).

In silico dose optimisation was conducted using 1,000 hypothetical SAM children and 1,000 hypothetical non-SAM children. Covariates (body-weight, age, and MUAC) of the SAM and non-SAM children was assumed to be uniformly distributed according to the observed value (Table 1). Day-7 lumefantrine concentrations, total exposures, and maximum lumefantrine concentrations of dose optimisation scenario were simulated and reported here. The final pharmacokinetic-pharmacodynamic model was also used for simulating treatment outcomes (i.e. 42 day cumulative malaria incidence) for the different dosing regimens. The pharmacodynamic simulations were performed using 100 replicates in order to calculate the 95% prediction intervals.

SUPPLEMENTARY FIGURES

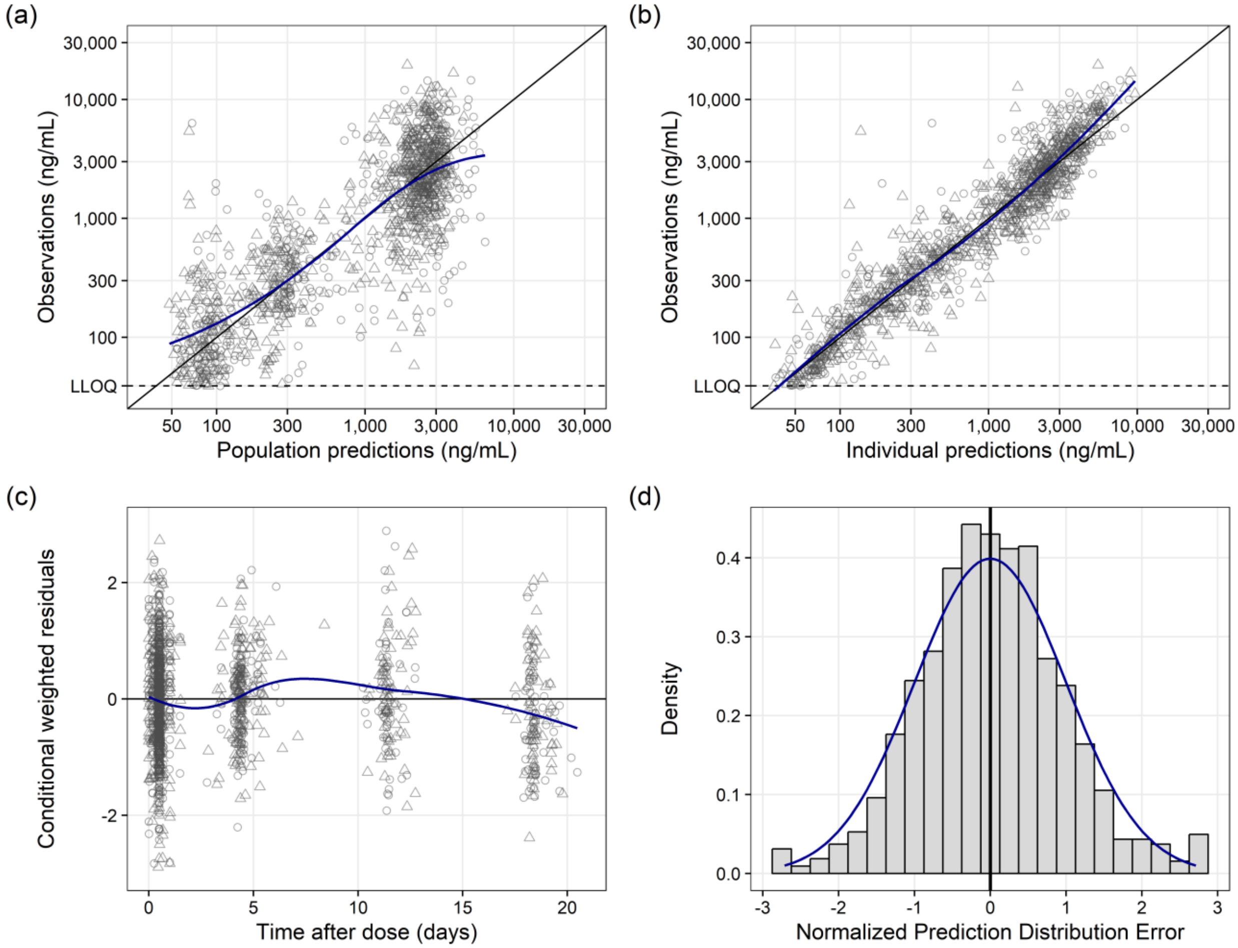

Figure S1. Goodness-of-fit plots of the final population pharmacokinetic model of lumefantrine.

(A) Population predictions versus observations, (B) Individual predictions versus observations, (C) time versus conditional weighted residuals, and (D) histogram of normalized prediction distribution errors. Open circles represent the observed lumefantrine data in non-SAM children and open triangles represent the observed lumefantrine data in SAM children. The trend lines represent the locally weighted least-square regressions based on the observations.

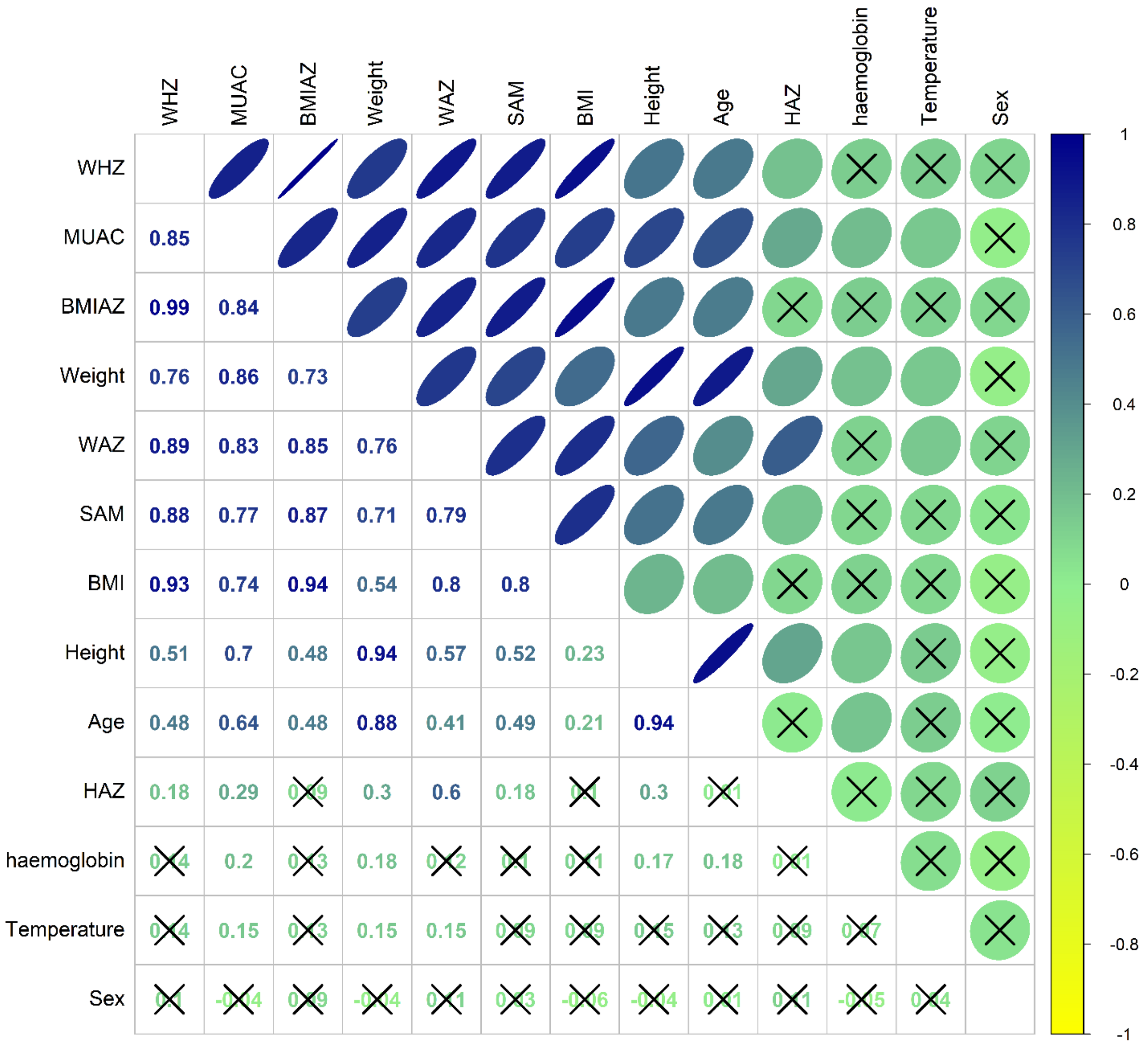

Figure S2. Covariate correlation plots.

Numbers represent the Pearson correlation coefficient between covariates. The "✕" represents non-significant correlations ($p > 0.01$). Abbreviations: BMI, body-mass-index; BMIAZ, BMI-for-age z-score; HAZ, height-for-age z-score; MUAC, mid-upper arm circumference; SAM, severe acute malnutrition status; WAZ, weight-for-age z-score; WHZ, weight-for-height z-score.

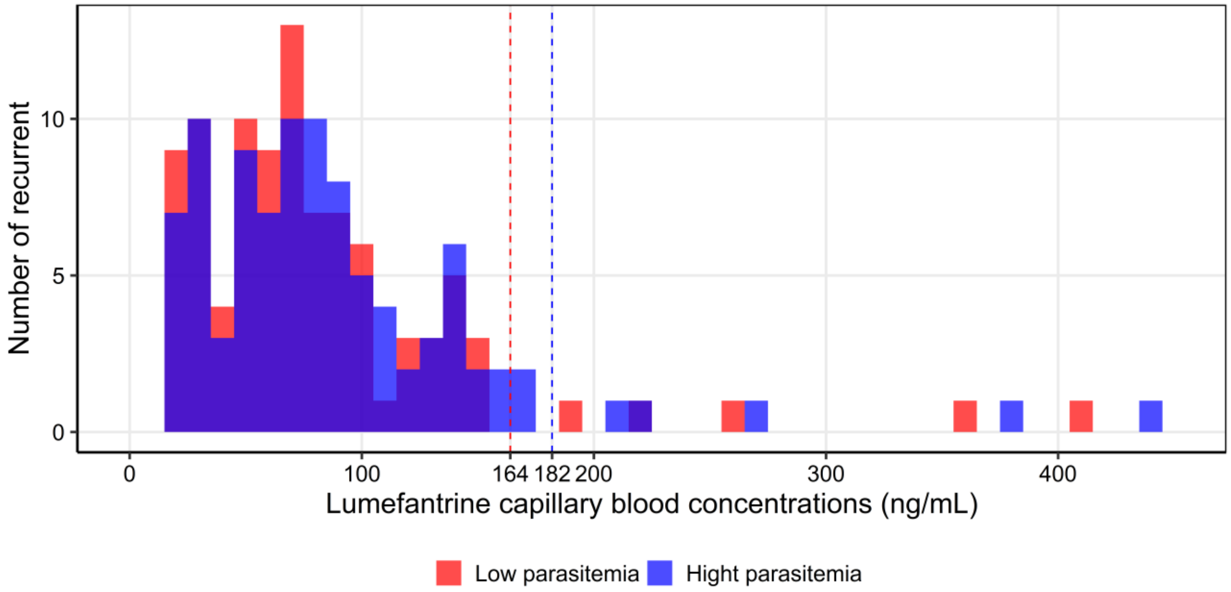

Figure S3. Histogram of the estimated minimum inhibitory concentration (MIC) of lumefantrine in children with recurrent malaria. Red bars represent the predicted lumefantrine concentrations using a low blood stage parasite density (~10^4) and blue bars represent the predicted lumefantrine concentrations using a high blood stage parasite density (~10^5). Vertical dashed lines represent the 95th percentile of lumefantrine concentrations under the two assumptions, assumed the lower and upper value of the clinical MIC of lumefantrine.

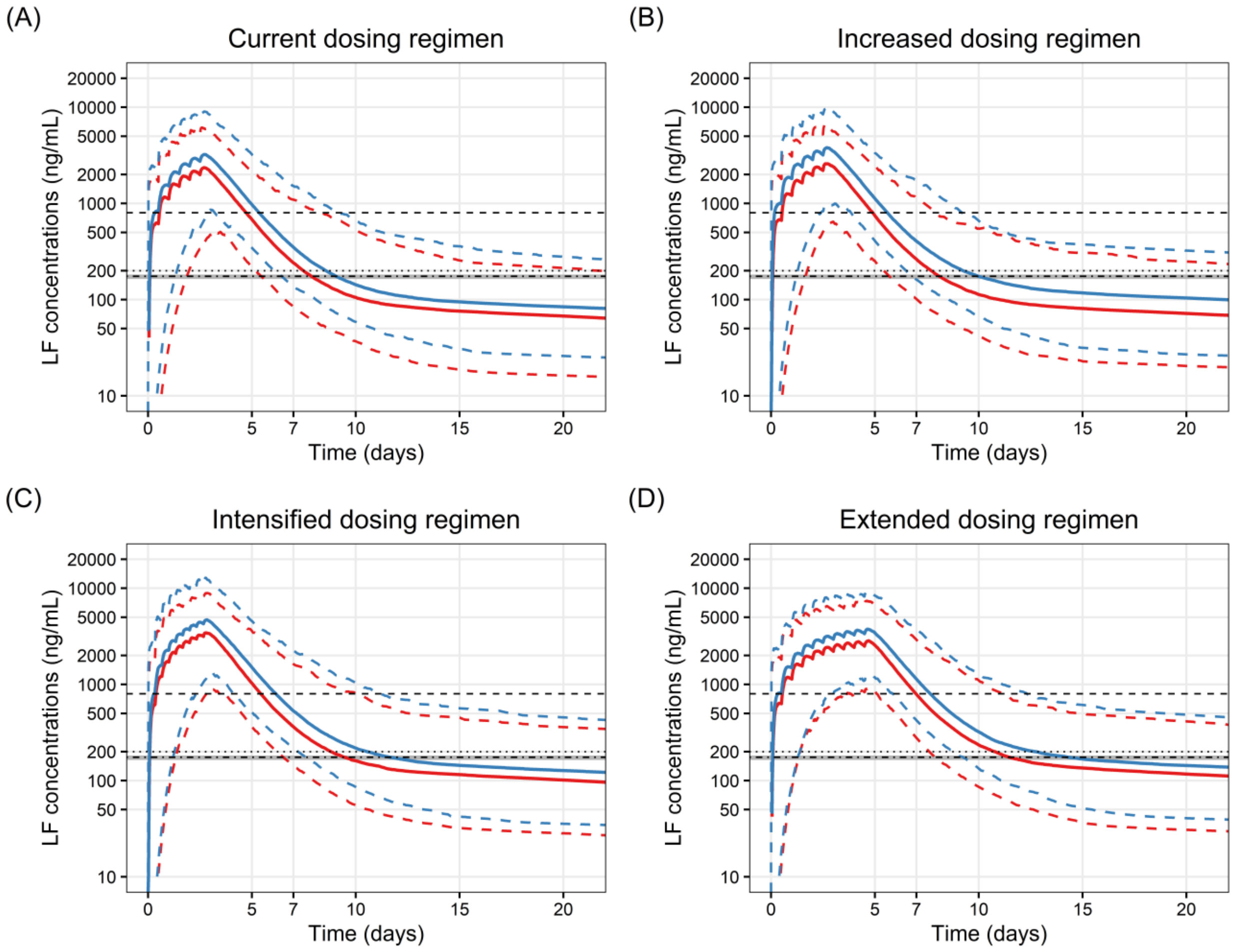

Figure S4. Simulated pharmacokinetic profiles in SAM (red) and non-SAM (blue) children receiving different dosing strategies.

Solid lines represent the median lumefantrine capillary blood concentration-time profiles, and the dashed lines represent the 95% prediction interval. Horizontal dashed lines represent the median day 7 lumefantrine concentration after standard dosing regimen in non-pregnant adult patients (801 ng/mL) (7). The dotted and dashed-dotted line represent the previously defined day 7 lumefantrine concentrations of 200 and 175 ng/mL (15, 16). Grey areas represent the estimated clinical MIC values in patients with recurrent malaria (164 ng/mL to 182 ng/mL).

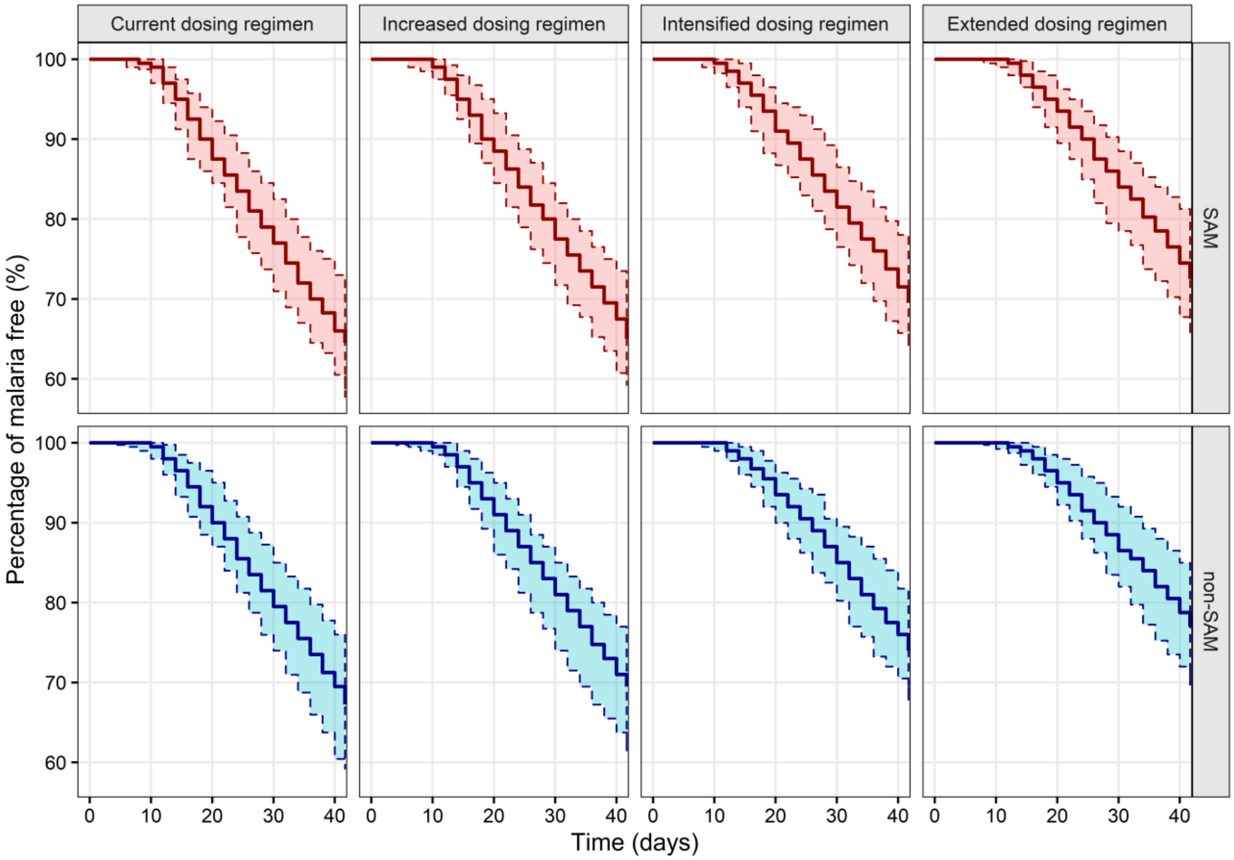

Figure S5. Simulated pharmacodynamic profiles in SAM (red) and non-SAM (blue) children receiving different dosing strategies.

Solid lines represent the predicted median survival estimate of the Kaplan-Meier plots and shaded areas represent the 95% prediction intervals.

SUPPLEMENTARY TABLE

Table S1. Simulated malaria incidence in SAM and non-SAM children receiving different dosing regimens of artemether-lumefantrine.

Regimens	SAM children	Non-SAM children
Predicted malaria-free incidence at day 20		
Current dosing regimen (120 mg LF every 12 hr for 3 days)	87.5% (84.5%, 92.3%)	90.0% (87.0%, 95.0%)
Increased dosing regimen (240 mg LF every 12 hr for 3 days)	88.5% (84.5%, 93.3%)	91.0% (86.0%, 95.0%)
Intensified dosing regimen (120 mg LF every 8 hr for 3 days)	91.0% (86.7%, 94.5%)	93.5% (90.0%, 96.3%)
Extended dosing regimen (120 mg LF every 12 hr for 5 days)	93.5% (89.5%, 96.3%)	95.0% (92.2%, 98.0%)
Predicted malaria-free incidence at day 42		
Current dosing regimen (120 mg LF every 12 hr for 3 days)	64.5% (57.7%, 71.5%)	67.3% (59.2%, 74.3%)
Increased dosing regimen (240 mg LF every 12 hr for 3 days)	65.0% (59.2%, 71.5%)	69.5% (61.5%, 75.3%)
Intensified dosing regimen (120 mg LF every 8 hr for 3 days)	69.5% (64.0%, 75.8%)	74.0% (67.7%, 80.3%)
Extended dosing regimen (120 mg LF every 12 hr for 5 days)	72.5% (65.5%, 79.3%)	77.0% (69.7%, 83.5%)

All value are reported as median (95% confidence interval).

Abbreviations: LF; lumefantrine, SAM; severe acute malnutrition.

SUPPLEMENTARY REFERENCES

(1) Keizer, R.J., van Benten, M., Beijnen, J.H., Schellens, J.H. & Huitema, A.D. Pirana and PCluster: a modeling environment and cluster infrastructure for NONMEM. *Comput Methods Programs Biomed* **101**, 72-9 (2011).

(2) Lindbom, L., Ribbing, J. & Jonsson, E.N. Perl-speaks-NONMEM (PsN)--a Perl module for NONMEM related programming. *Comput Methods Programs Biomed* **75**, 85-94 (2004).

(3) Jonsson, E.N. & Karlsson, M.O. Xpose--an S-PLUS based population pharmacokinetic/pharmacodynamic model building aid for NONMEM. *Comput Methods Programs Biomed* **58**, 51-64 (1999).

(4) Ahn, J.E., Karlsson, M.O., Dunne, A. & Ludden, T.M. Likelihood based approaches to handling data below the quantification limit using NONMEM VI. *J Pharmacokinet Pharmacodyn* **35**, 401-21 (2008).

(5) Savic, R.M., Jonker, D.M., Kerbusch, T. & Karlsson, M.O. Implementation of a transit compartment model for describing drug absorption in pharmacokinetic studies. *J Pharmacokinet Pharmacodyn* **34**, 711-26 (2007).

(6) Petersson, K.J., Hanze, E., Savic, R.M. & Karlsson, M.O. Semiparametric distributions with estimated shape parameters. *Pharm Res* **26**, 2174-85 (2009).

(7) Kloprogge, F. *et al.* Artemether-lumefantrine dosing for malaria treatment in young children and pregnant women: A pharmacokinetic-pharmacodynamic meta-analysis. *PLoS Med* **15**, e1002579 (2018).

(8) Mandema, J.W., Verotta, D. & Sheiner, L.B. Building population pharmacokinetic--pharmacodynamic models. I. Models for covariate effects. *J Pharmacokinet Biopharm* **20**, 511-28 (1992).

(9) Anderson, B.J. & Holford, N.H. Mechanistic basis of using body size and maturation to predict clearance in humans. *Drug Metab Pharmacokinet* **24**, 25-36 (2009).

(10) WHO Multicentre Growth Reference Study Group. *WHO Child Growth Standards: Length/height-for-age, weight-for-age, weight-for-length, weight-for-height and body mass index-for-age: Methods and development.* (World Health Organization: Geneva, Switzerland, 2006).

(11) Chotsiri, P. *et al.* Optimal dosing of dihydroartemisinin-piperaquine for seasonal malaria chemoprevention in young children. *Nature communications* **10**, 480 (2019).

(12) Bergstrand, M., Nosten, F., Lwin, K.M., Karlsson, M.O., White, N.J. & Tarning, J. Characterization of an in vivo concentration-effect relationship for piperaquine in malaria chemoprevention. *Science translational medicine* **6**, 260ra147 (2014).

(13) Savic, R.M. & Karlsson, M.O. Importance of shrinkage in empirical bayes estimates for diagnostics: problems and solutions. *AAPS J* **11**, 558-69 (2009).

(14) Bergstrand, M., Hooker, A.C., Wallin, J.E. & Karlsson, M.O. Prediction-corrected visual predictive checks for diagnosing nonlinear mixed-effects models. *AAPS J* **13**, 143-51 (2011).

(15) Price, R.N. *et al.* Molecular and pharmacological determinants of the therapeutic response to artemether-lumefantrine in multidrug-resistant Plasmodium falciparum malaria. *Clin Infect Dis* **42**, 1570-7 (2006).

(16) WorldWide Antimalarial Resistance Network (WWARN) Lumefantrine PK/PD Study Group. Artemether-lumefantrine treatment of uncomplicated Plasmodium falciparum malaria: a systematic review and meta-

analysis of day 7 lumefantrine concentrations and therapeutic response using individual patient data. *BMC Med* **13**, 227 (2015).

(17) Tchaparian, E. *et al.* Population Pharmacokinetics and Pharmacodynamics of Lumefantrine in Young Ugandan Children Treated With Artemether-Lumefantrine for Uncomplicated Malaria. *J Infect Dis* **214**, 1243-51 (2016).

(18) Ezzet, F., van Vugt, M., Nosten, F., Looareesuwan, S. & White, N.J. Pharmacokinetics and pharmacodynamics of lumefantrine (benflumetol) in acute falciparum malaria. *Antimicrob Agents Chemother* **44**, 697-704 (2000).
